# The assessment of serum-mediated phagocytosis of necrotic material by polymorphonuclear leukocytes to diagnose and predict the clinical features of systemic lupus erythematosus: an observational longitudinal study

**DOI:** 10.1186/s13075-016-0941-1

**Published:** 2016-02-10

**Authors:** Michele Compagno, Birgitta Gullstrand, Søren Jacobsen, Gro Ø. Eilertsen, Jan Åke Nilsson, Christian Lood, Andreas Jönsen, Lennart Truedsson, Gunnar Sturfelt, Anders A. Bengtsson

**Affiliations:** Department of Clinical Sciences, Section of Rheumatology, Lund University, Lund, Sweden; Department of Rheumatology, Rigshospitalet, Copenhagen University Hospital, Copenhagen, Denmark; Department of Clinical Medicine, Bone and Joint Research Group, Faculty of Health Science, University of Tromsø, Tromsø, Norway; Department of Laboratory Medicine, Section of Microbiology, Immunology and Glycobiology, Lund University, Lund, Sweden

**Keywords:** Systemic lupus erythematosus, Biomarkers, Diagnostic accuracy, Flow cytometry, Disease activity, Lupus nephritis

## Abstract

**Background:**

Serum-mediated phagocytosis of antibody- and complement-opsonized necrotic cell material (NCM) by polymorphonuclear leukocytes can be quantified by using a flow cytometry–based assay. The phagocytosis of necrotic cell material (PNC) assay parallels the well-known lupus erythematosus cell test. In this study, we aimed to investigate the diagnostic accuracy of the assay and the relationship with clinical manifestations and disease activity in systemic lupus erythematosus (SLE).

**Methods:**

The diagnostic accuracy for SLE diagnosis of the PNC assay was studied by cross-sectional assessment of blood samples from 148 healthy control subjects and a multicenter rheumatic group (MRG) of 529 patients with different rheumatic symptoms. A cohort of 69 patients with an established SLE diagnosis (SLE cohort) underwent longitudinal clinical and laboratory follow-up for analysis of the temporal relationships between PNC positivity and specific clinical manifestations.

**Results:**

In 35 of 529 MRG patients, 13 of whom had SLE, the PNC assay result was positive. Combined positivity of the PNC assay and anti–double-stranded DNA antibodies increased specificity and positive predictive value for SLE diagnosis to 0.99 and 0.67, respectively. In the longitudinal study, 42 of 69 SLE cohort patients had positive results in the PNC assay at least once. PNC assay positivity was associated with current hematological manifestations and could predict mucocutaneous manifestations. When combined with hypocomplementemia, PNC positivity preceded increased Systemic Lupus Erythematosus Disease Activity Index 2000 score, glomerulonephritis, and alopecia.

**Conclusions:**

Serum-mediated PNC by polymorphonuclear leukocytes is commonly but not exclusively seen in patients with SLE. The PNC assay may be used in follow-up of patients with SLE and, especially in combination with other routinely assessed laboratory tests, may help to predict flares and different clinical manifestations, including glomerulonephritis. Our results encourage further development of the PNC assay as a complementary laboratory tool in management of patients with SLE.

## Background

Systemic lupus erythematosus (SLE) is an autoimmune syndrome of unknown etiology, with a complex interplay between genetic and environmental factors [1, 2] and between the innate and the adaptive immune systems [3]. The disease commonly affects women of childbearing age and is characterized by chronic inflammation in several different organ systems with potentially life-threatening outcomes. Many clinical manifestations of SLE are caused by immune complex deposition in target organ and subsequent inflammation caused by complement activation and infiltration of immune cells. Among the different cells involved in the pathogenesis of SLE, the crucial role played by polymorphonuclear leukocytes (PMNs) has recently been emphasized [4–12]. The immune complexes mostly contain nuclear material able to stimulate immune cells through Toll-like receptor 7 (TLR7) and TLR9, and are formed upon binding of autoantibodies to different antigens, often remnants of cells dying due to impaired clearance of apoptotic cells [8, 13].

Although SLE classification criteria have been formulated and periodically updated [14–17], these criteria are constructed to classify patient materials for research purposes and are not diagnostic criteria meant to be used in clinical practice [18]. Furthermore, there is a great need for novel diagnostic and prognostic tools to complement the ones already in use, including assessment of antibodies against double-stranded DNA (dsDNA) and histone proteins, as well as consumption of components of the classical pathway of the complement system. The first laboratory test proposed as a diagnostic tool for SLE was the lupus erythematosus (LE) cell, described in 1948 as a specific finding in bone marrow leukocytes in patients affected by SLE [19]. Further studies have shown that LE cells consist of mature PMNs in which the nucleus has been dislocated to the periphery of the cell after engulfment of antibody- and complement-opsonized nuclear material [20, 21]. The presence of LE cells was included in the list of the classification criteria for SLE [14, 17] and related to more severe clinical manifestations [22, 23]. The LE cell was rarely observed in other rheumatic [24] and non-rheumatic disorders [25], but published data on the diagnostic accuracy of the LE cell test in unselected patients are still missing [22, 26, 27]. Previous studies showed that the phenomenon is dependent on anti-dsDNA antibodies [21] and anti-histone antibodies, in particular anti-histone H1 antibodies [28]. Because of its complexity, the assessment of LE cells by light microscopy was abandoned as a routine test in favor of other diagnostic tools, including anti-dsDNA antibody analysis.

More recently, a flow cytometry–based assay—the phagocytosis of necrotic cells (PNC) assay—was developed as an in vitro assessment and quantification of LE cells in patients with suspected SLE [29], but its use in clinical practice has never been validated. In a previous study [30], our group demonstrated that the outcome of the PNC assay is often positive in patients with SLE with increased disease activity. The phagocytosis seems to be associated with oxidative burst activity that is mediated by Fc γ-receptor IIA (FcγRIIA), FcγRIIIB, and complement receptor type 1 in combination. The phagocytosis is more efficient in the presence of high levels of different anti-histone antibodies and when the classical pathway of the complement system is functional and active [30].

In this study, we show that the PNC assay, by detecting serum-mediated phagocytosis of necrotic cell material (NCM), may be used as a tool in the process of diagnosing SLE. Furthermore, we found that the outcome of the PNC assay was related to clinical manifestations and, in combination with other established biomarkers, with disease activity.

## Methods

### Assay for phagocytosis of necrotic cell material assay

Serum-mediated phagocytosis of NCM by PMNs was performed as described by Böhm [29], with some modifications [30]. Briefly, PMNs and peripheral blood mononuclear cells (PBMCs) were isolated from heparinized blood using Polymorphprep™ (Axis-Shield Poc AS, Oslo, Norway) according to the manufacturer’s protocol. To induce cell death, PBMCs were incubated for 10 minutes at 70 °C, and the NCM was stained with propidium iodide (PI; BD Biosciences, San Diego, CA, USA). PMNs were stained with anti-CD66 fluorescein isothiocyanate antibodies (Dako A/S, Glostrup, Denmark).

**Fig. 1:**
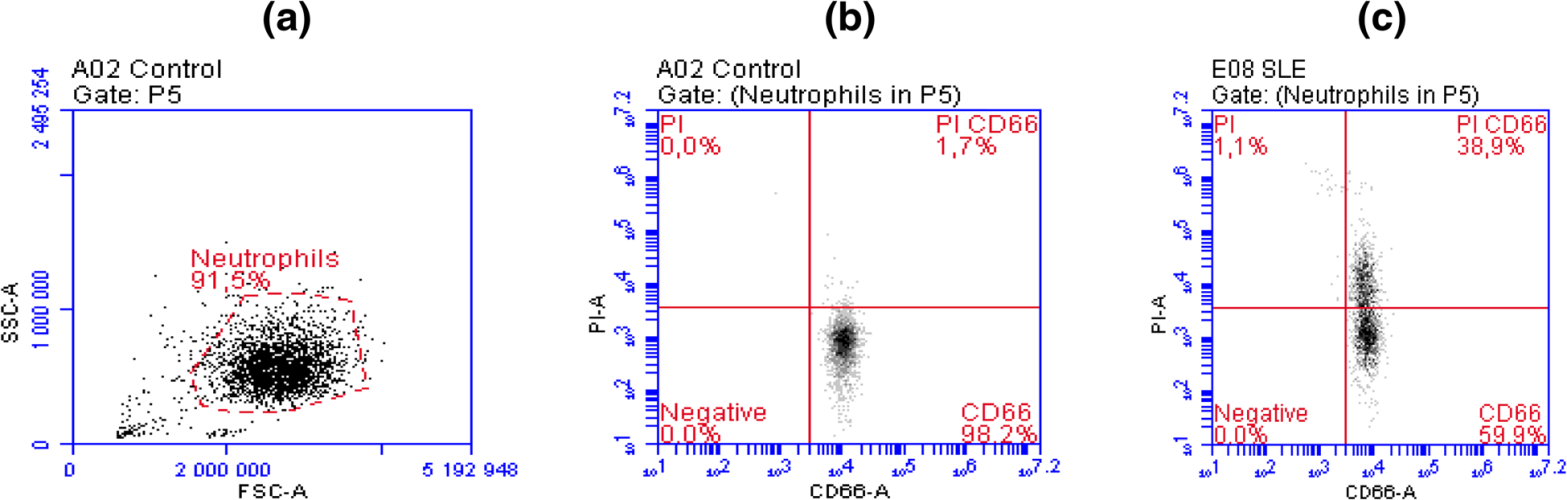
Flow cytometric analysis results. **a** Forward scatter analysis (FSC-A) and side scatter analysis (SSC-A) of polymorphonuclear cells (neutrophils in gate P5) in a healthy control. **b** Negative outcome of phagocytosis of necrotic cell material (PNC) assay in a healthy control, where only 1.7 % of gated neutrophils are positive for propidium iodide (PI) and CD66. **c** Positive outcome of PNC assay in a patient affected by systemic lupus erythematosus, where 38.9 % of gated neutrophils are positive for PI and CD66

For autoantibody binding and complement activation, PI-labeled NCM (4.5 × 10^5^ cells) was incubated with 30 μl of undiluted serum at room temperature for 20 minutes, followed by addition of PMNs isolated from healthy individuals (0.3 × 10^6^ cells in a total volume of 300 μl) for another incubation at 37 °C for 15 minutes. Cells were washed with phosphate-buffered saline, pH 7.2, containing 0.1 % human serum albumin (Sigma-Aldrich, St. Louis, MO, USA) before analysis by flow cytometry. PMNs were identified on the basis of forward and side scatter properties and by computerized gating (Fig. 1). Phagocytosis was calculated from the percentage of cells positive for both CD66 and PI (percentage of CD66+PI+ PMNs). A sample was considered positive if the PNC value was higher than the mean value obtained in 148 healthy individuals added with 3 standard deviations. All the measurements performed in the SLE cohort were included to analyze the relationships between PNC results and SLE-related clinical manifestations. Only the result obtained in the first measurement in every patient in the SLE cohort was taken into account for the comparison with the outcome of PNC assay obtained in healthy individuals and in patients from the multicenter rheumatic group (MRG).

### Other laboratory tests

In the SLE cohort, routine laboratory tests were used for the longitudinal measurement of all the variables needed to assess disease activity according to the Systemic Lupus Erythematosus Disease Activity Index 2000 (SLEDAI-2K) [31], including the complement components C1q, C3, and C4. A semi-quantitative assessment of anti-nuclear antibodies (ANA) and anti-dsDNA antibodies [*Crithidia luciliae* immunofluorescence test (CLIFT)] was performed with immunofluorescence tests, using as substrates Hep-2 cells and *Crithidia luciliae*, respectively (Euroimmun, Lübeck, Germany). Enzyme-linked immunosorbent assay measurement of antibodies against a mixture of histone proteins was performed as previously described [30]. Only ANA and CLIFT were assessed cross-sectionally in the MRG patients, by the routinely used method in the respective participating center, as described elsewhere [32].

### Patients and controls

**Fig. 2:**
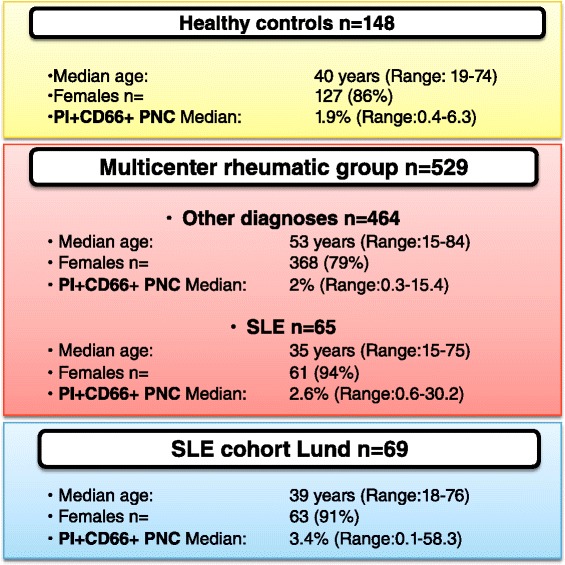
Demographics and results of phagocytosis of necrotic cell material (PNC) assay in all the study participants. Age of the patients and percentage of double-positive polymorphonuclear cells (PI+CD66+ PMNs) are expressed as median values (range in brackets). The percentage of double-positive PNC results is significantly higher in patients affected by SLE compared with healthy controls and non-SLE patients in the multicenter rheumatic group. *PI* propidium iodide, *SLE* systemic lupus erythematosus

Demographics of the 148 healthy volunteers and 529 MRG patients are presented in Fig. 2. In three different centers [Copenhagen in Denmark (138 patients), Lund in Sweden (269 patients), and Tromsø in Norway (122 patients)], MRG patients were recruited and grouped according to initial clinical diagnoses, as previously described [32]. In brief, patients with recent onset of any suspected rheumatic disease and referred for the first time to rheumatologists were recruited for clinical assessment and analysis of ANA and anti-dsDNA antibodies. The cross-sectional performance of the PNC assay in these patients was intended to obtain reference values of the test in rheumatic patients and to evaluate its diagnostic value in a realistic clinical situation, such as in patients with early onset of rheumatic manifestations, when the diagnosis is still unknown. We focused on the outcome of the test in the subgroup of patients later diagnosed with SLE, in comparison with other groups of rheumatic diseases. The final diagnoses were formulated by the clinician at the participating center on the basis of the clinical features, regardless of the fulfillment of classification criteria, after a median follow-up period of 4.7 years (Table 1).

**Table 1:** Positive outcome of PNC assay and CLIFT in MRG patients

Diagnosis	PNC+ (%)	CLIFT+ (%)	PNC+CLIFT+ (%)	Total (%)
Arthralgia [%]	2 (5.7) [5.9]	0	0	34 (6.4) [100]
Connective tissue disease [%]	3 (8.6) [5.6]	7 (15.9) [13]	2 (13.3) [3.7]	54 (10.2) [100]
Dermatological disorder [%]	0	0	0	17 (3.2) [100]
Inflammatory joint disease [%]	3 (8.6) [2.7]	8 (18.2) [7.1]	2 (13.3) [1.8]	112 (21.2) [100]
Osteoarthritis [%]	6 (17.1) [10.2]	0	0	59 (11.2) [100]
Non-rheumatic disease [%]	6 (17.1) [5]	6 (13.6) [5]	1 (6.7) [0.8]	120 (22.7) [100]
Soft tissue rheumatism [%]	1 (2.9) [4]	0	0	25 (4.7) [100]
Systemic inflammatory disease [%]	1 (2.9) [4.2]	2 (4.5) [8.3]	0	24 (4.5) [100]
Unspecified [%]	0	0	0	19 (3.6) [100]
SLE [%]	13 (37.1) [20]	21 (47.7) [32.3]	10 (66.7) [15.4]	65 (12.3) [100]
Total	35 (100)	44 (100)	15 (100)	529 (100)

*CLIFT Crithidia luciliae* immunofluorescence test, *PNC* phagocytosis of necrotic cell material, *SLE* systemic lupus erythematosus

The different subsets of patients are grouped on the basis of final diagnosis recorded after a median of 4.7 years, as described elsewhere [32]. The percentage of positive laboratory tests is reported in round brackets; the percentage of positive patients in the different subsets is reported in square brackets

To study the temporal associations between the outcome of the PNC assay and SLE-related clinical phenotypes, we performed the PNC assay repeatedly over time in a cohort of 69 patients (Fig. 2) affected by SLE who fulfilled the American College of Rheumatology (ACR) 1982 classification criteria for SLE [17]. These patients participated in a prospective follow-up program at the Rheumatology Clinic, University Hospital, Lund, Sweden, with the general purpose of improving care of patients with SLE and to identify clinical and laboratory variables that could be considered as markers or predictors of complications and exacerbations of the disease. They were followed longitudinally with a median of 14 (2–43) visits periodically scheduled every 60 ± 20 days. An extensive set of clinical and laboratory variables were registered in a database tailored for the study. Serum samples were collected before and after the date of the clinical assessment when needed.

Demographics, clinical features, and prevalence of manifestations included in the ACR classification criteria in SLE cohort are summarized in Table 2. Disease activity was assessed every time, using the SLEDAI-2K [31]. For the assessment of relationships with outcomes of the PNC assay in combination with other biomarkers, a modified version of the SLEDAI-2K was used, excluding any score given for low levels of complement factors and/or anti-dsDNA antibodies.

**Table 2:** Demographics, clinical characteristics, and outcomes of PNC assay in 69 patients with SLE (SLE cohort)

Characteristics	Data
Age, yr	
Mean (SD)	41.4 (13.7)
Median (range)	39.2 (18–76)
Female sex, *n* (%)	63 (91.3)
Disease duration, yr, median (range)	7.5 (0–41)
Follow-up duration, d, median (range)	778 (139–1792)
Corticosteroid treatment, *n* (%)	33 (47.8)
Prednisolone equivalent, average dose last 2 mo, median (range)	10 (1–60) mg/d
Antimalarial treatment	46 (66.7)
Hydroxychloroquine, average dose last 2 mo, median (range)	200 (46.6–400) mg/d
Immunomodulatory treatment, *n* (%)	37 (53.6)
ACR classification criteria *n* (%)	
Malar rash	46 (67 %)
Discoid rash	18 (26 %)
Photosensitivity	42 (61 %)
Oral ulcer	16 (23 %)
Arthritis	55 (80 %)
Serositis	35 (51 %)
Glomerulonephritis	35 (51 %)
Hematologic disorder	38 (55 %)
Neurologic disorder	3 (4 %)
Immunologic disorder	53 (77 %)
ANA positivity	69 (100 %)
Outcome of PNC assay, % CD66+PI+ PMNs	
Mean (SD)	7.3 (9.0)
Median (range)	3.4 (0.8–49.9)

*ACR* American College of Rheumatology, *ANA* anti-nuclear antibodies; *SD* standard deviation, *CD66+PI+ PMNs* double-stained polymorphonuclear cells, *PI* propidium iodide, *PNC* phagocytosis of necrotic cell material

### Ethics

All subjects entered the study after giving informed written consent, according to the Helsinki declaration. The study was performed according to the approval by the local ethical committees of the participating centers in Lund, Sweden (Research Ethics Committee, Medical Faculty, Lund University); Tromsø, Norway (Regional Committee for Medical and Health Research Ethics - REC North); and Copenhagen, Denmark (research ethics committees for Copenhagen and Frederiksberg).

### Statistical analysis

Descriptive statistics were calculated, and the Kruskal-Wallis test was used for analysis of non-parametric data. Sensitivity, specificity, positive predictive value (PPV), likelihood ratio for positive (LR+) and negative (LR-) result of PNC assay, CLIFT and the combination of the two assays (PNC+CLIFT+) were calculated to assess their diagnostic accuracy for SLE diagnosis in MRG patients. Moreover, receiver operating characteristic (ROC) curves were plotted and area under the curve (AUC) were calculated for PNC assay, CLIFT, the combination of the two assays (PNC+CLIFT+) and ANA. Temporal associations between positive outcomes of PNC assays and relevant clinical phenotypes were evaluated with a generalized linear mixed-effects model (PROC GENMOD). Odds ratios (ORs), 95 % confidence intervals (CIs), and statistical significance levels (*p* values) were calculated. A *p* value ≤0.05 defined statistical significance. SAS 9.3 (SAS Institute, Cary, NC, USA) and IBM SPSS Statistics 20.0 (IBM, Armonk, NY, USA) software was used for all statistical analyses. Only visits within intervals of 60 ± 20 days were considered for temporal associations.

## Results

Positivity in PNC assays is specific but not exclusively found in patients with SLE. To assess its diagnostic accuracy for SLE diagnosis, the PNC assay was performed in the 529 MRG patients. The outcomes of PNC assays in MRG patients are summarized in Table 1. The PNC assay was positive in 35 MRG patients (6.6 %) with recent onset of any rheumatic symptoms. Among these patients, the most common clinical diagnosis was SLE (13 cases), resulting in a sensitivity of the test at 0.20, specificity of the test at 0.95, and PPV of the test for SLE diagnosis at 0.37. Other, less common diagnoses in PNC-positive MRG patients were arthralgia (two cases, 5.9 % of MRG patients affected by arthralgia and 5.7 % of PNC-positive patients), osteoarthritis (six cases, 10.2 % of MRG patients affected by osteoarthritis and 17.1 % of PNC-positive patients), inflammatory joint disease (three cases, 2.7 % of MRG patients affected by inflammatory joint disease and 8.6 % of PNC-positive patients), non-rheumatic disease (six cases, 5 % of MRG patients affected by non-rheumatic disease and 17.1 % of PNC-positive patients), connective tissue disease (three cases, 5.6 % of MRG patients affected by connective tissue disease and 8.6 % of PNC-positive patients), systemic inflammatory disease (one case, 4.2 % of MRG patients affected by systemic inflammatory disease and 2.9 % of PNC-positive patients), and soft tissue rheumatism (one case, 4 % of MRG patients affected by soft tissue rheumatism and 2.9 % of PNC-positive patients). In the subgroup of 44 anti-dsDNA–positive MRG patients, 15 (34.1 %) had a positive PNC test result. Among them, 10 had SLE, 2 had a connective tissue disease, 2 had an inflammatory joint disease, and 1 had a non-rheumatic disease. The positivity of CLIFT alone resulted in a sensitivity at 0.32, specificity at 0.95, and PPV at 0.48. The combined positivity of CLIFT and the PNC assay increased the specificity to 0.99 and the PPV to 0.67 in patients with SLE (Table 3).

**Table 3:** Diagnostic accuracy for SLE of the PNC assay, CLIFT, and their combined positivity in MRG patients

	PNC+	CLIFT+	PNC+CLIFT+
Sensitivity (95 % CI)	0.20 (0.12–0.37)	0.32 (0.22–0.44)	0.15 (0.09–0.26)
Specificity (95 % CI)	0.95 (0.93–0.97)	0.95 (0.93–0.97)	0.99 (0.97–0.99)
PPV (95 % CI)	0.37 (0.23–0.54)	0.48 (0.34–0.62)	0.67 (0.42–0.85)
LR+ (95 % CI)	4.22 (2.24–7.96)	6.52 (3.83–11.10)	14.28 (5.04–40.46)
LR− (95 % CI)	0.84 (0.74–0.95)	0.71 (0.60–0.84)	0.86 (0.77–0.95)

*CI* confidence interval, *PPV* positive predictive value, *LR+* likelihood ratio for positive result, *LR−* likelihood ratio for negative result, *CLIFT Crithidia luciliae* immunofluorescence test, *PNC* phagocytosis of necrotic cell material, *MRG* multicenter rheumatic group, *SLE* systemic lupus erythematosus

**Fig. 3:**
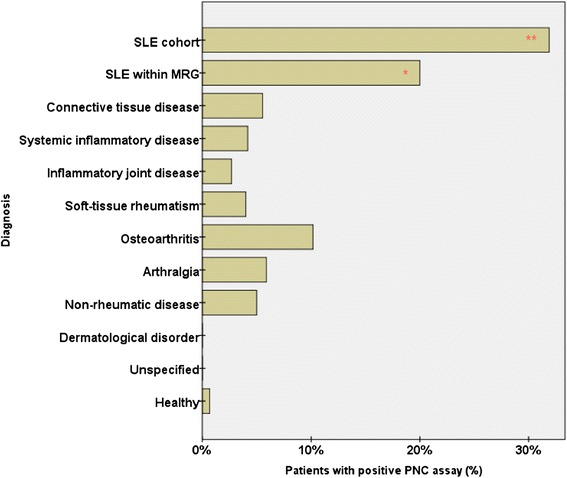
Percentage of multicenter rheumatic group (MRG) patients and healthy controls with positive outcomes of phagocytosis of necrotic cell material (PNC) assays. Within the subgroups of patients with systemic lupus erythematosus (SLE), the percentage of patients with positive PNC assay results was significantly higher than in other subgroups of patients and in healthy controls. **Strongly significant difference compared with other subsets of patients (*p* < 0.01). *Significant difference compared with other subsets of patients (*p* < 0.05)

Overall, the positive outcomes of PNC assays were seen mostly in patients with SLE. The percentages of PNC-positive patients in the different subgroups and in healthy individuals are reported in Fig. 3.

**Fig. 4:**
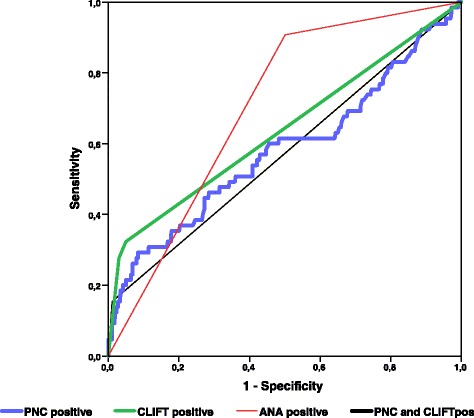
Area under the ROC curve analysis showing diagnostic accuracy of different tests for systemic lupus erythematosus in multicenter rheumatic group patients. *ANA* anti-nuclear antibodies, *CLIFT Crithidia luciliae* immunofluorescence test, *PNC* phagocytosis of necrotic cell material

Area under the ROC curve analysis plotting the diagnostic accuracy for SLE of PNC assay and its combination with CLIFT, in comparison with ANA and CLIFT alone, is shown in Fig. 4 and Table 4. ANA and CLIFT had the best AUC (0.7 and 0.64, respectively), suggesting a sharper and more immediate diagnostic role than PNC assay for SLE, in patients with recent onset of rheumatic symptoms.

**Table 4:** Positive results for PNC assay, CLIFT, ANA, and combination of PNC and CLIFT

Test result	Area under the curve	95 % confidence interval	
		Lower bound	Upper bound
PNC+	0.58	0.50	0.66
CLIFT+	0.64	0.56	0.72
ANA+	0.70	0.65	0.76
PNC+CLIFT+	0.57	0.49	0.65

*ANA* anti-nuclear antibodies, *CLIFT Crithidia luciliae* immunofluorescence test, *PNC* phagocytosis of necrotic cell material

### Relationship between PNC positivity over time and clinical manifestations in patients with SLE

Significant differences were found in the prevalence of clinical manifestations in patients with SLE, grouped on the basis of the outcome of PNC assay over time (Table 5). On one hand, 12 patients with SLE had persistent PNC positivity during the entire follow-up period on the basis of 166 total assessments. On the other hand, 31 patients with SLE had persistently negative PNC assay results in a total of 475 assessments. The remaining 459 assessments in 26 patients with SLE displayed variable PNC results over time.

**Table 5:** Most frequent clinical manifestations in patients with SLE

Clinical manifestation	Always positive PNC (*n* = 12 patients, 166 assessments)	Always negative PNC (*n* = 31 patients, 475 assessments)	Variable PNC (*n* = 26 patients, 459 assessments)
Glomerulonephritis	31.9 %	10.5 %^a^	6.5 %^a^
Arthritis	1.8 %^b^	16.6 %	6.1 %^a,b^
Mucocutaneous	24.7 %	21.3 %	13.9 %^a,b^
Alopecia	10.8 %	7.8 %	7.2 %
Hematologic	3.0 %^c^	1.1 %^c^	7.0 %^b^
SLEDAI-2K >1	57.8 %	48.8 %	44.7 %^a,b^

*SLEDAI-2K* Systemic Lupus Erythematosus Disease Activity Index 2000

^a^Significant difference (*p* ≤ 0.05) compared with “Always positive PNC”

^b^Significant difference (*p* ≤ 0.05) compared with “Always negative PNC”

^c^Significant difference (*p* ≤ 0.05) compared with “Variable PNC”

The 69 patients are grouped depending on the outcome of PNC assay (positive or negative) over time

Glomerulonephritis was significantly more prevalent among patients with persistent PNC positivity, whereas arthritis was much more prevalent among those with persistently negative test results. Alopecia, mucocutaneous features, and increased SLEDAI-2K scores were more prevalent in patients with persistent PNC positivity, but the difference was not statistically significant compared with the patients with consistently negative PNC results.

### Temporal relationships between PNC positivity and occurrence of clinical manifestations

In the SLE cohort from Lund, the PNC assay showed a significant correlation (*p* ≤ 0.05) with different clinical manifestations. In particular, a positive PNC test result was associated with active hematologic features such as leukopenia and thrombocytopenia (OR 7.67, 95 % CI 2.98–19.74). Positivity in the PNC assay was able to predict mucocutaneous manifestations within the next 60 ± 20 days (OR 1.68, 95 % CI 1.15–2.46), whereas it displayed an inverse association with future flare of active arthritis (OR 0.36, 95 % CI 0.16–0.82). No significant temporal correlations between PNC assay outcomes and increased SLEDAI-2K scores were found (Table 6).

**Table 6:** Temporal associations between occurrence of the most frequent clinical manifestations and positive PNC assay results

Clinical manifestation	Occurrence of clinical manifestation					
	Same time as positive PNC			After positive PNC		
	OR	95 % CI	*p* Value	OR	95 % CI	*p* Value
Glomerulonephritis	1.48	0.95–2.31	0.080	1.41	0.80–2.50	0.233
Arthritis	0.72	0.39–1.34	0.303	0.36	0.16–0.82	0.015
Mucocutaneous	1.36	0.94–1.97	0.105	1.68	1.15–2.46	0.007
Alopecia	0.87	0.47–1.60	0.657	1.37	0.81–2.32	0.239
Hematological features	7.67	2.98–19.74	<0.001	0.59	0.28–1.26	0.173
SLEDAI-2K >0	1.27	0.86–1.88	0.222	1.24	0.83–1.85	0.288

*OR* odds ratio, *CI* confidence interval, *PNC* phagocytosis of necrotic cell material, *SLEDAI-2K* Systemic Lupus Erythematosus Disease Activity Index 2000

### Combination of PNC assay with other laboratory tests correlates with clinical manifestations

The PNC assay relies on the presence of autoantibodies, in particular antibodies directed against histone proteins and dsDNA, as well as an intact classical pathway of the complement system. We therefore wanted to analyze the relationships between different clinical manifestations and positive PNC assay results in combination with the presence of antibodies against dsDNA or histones or with low levels of complement components C1q, C3, and C4.

An isolated presence of anti-dsDNA antibodies in our SLE cohort had significant associations with glomerulonephritis, mucocutaneous manifestations, alopecia, and increased SLEDAI-2K score (data not shown). The presence of anti-dsDNA antibodies together with a positive PNC assay result showed no association with any current or upcoming clinical manifestation.

Positivity of anti-histone antibodies alone was associated with active glomerulonephritis and mucocutaneous manifestations. Anti-histone antibodies in combination with a positive PNC test result could predict future flare of mucocutaneous manifestations within 2 months (OR 2.33, 95 % CI 1.21–4.78, *p* = 0.011).

Low levels of C1q were associated with concomitant glomerulonephritis. Low levels of C3 (with or without concomitant low levels of C4) were associated with mucocutaneous manifestations (e.g., rash, oral ulcers). The combination of hypocomplementemia with a positive PNC assay showed a strong relationship with active glomerulonephritis, alopecia, and increased modified SLEDAI-2K score. In particular, a positive PNC assay result in combination with a low level of C3 was associated with increased SLEDAI-2K score (OR 2.34, 95 % CI 1.29–4.24, *p* = 0.005), predicted flare of glomerulonephritis (OR 2.29, 95 % CI 1.12–4.67, *p* = 0.023), and alopecia (OR 5.64, 95 % CI 1.49–21.32, *p* = 0.011) within the next 2 months. Low levels of C1q in combination with positive PNC assay results were associated with present (OR 1.74, 95 % CI 1.01–2.99, *p* = 0.045) and upcoming (OR 2.12, 95 % CI 1.03–4.37, *p* = 0.042) active glomerulonephritis.

## Discussion

Our data show that the PNC assay might partially improve the diagnostic accuracy of anti-dsDNA antibodies for SLE in patients with a recent onset of rheumatic symptoms. In patients with established SLE, its positive outcome was strongly correlated with ongoing hematological features such as leukopenia and thrombocytopenia. Furthermore, by itself and/or in combination with other laboratory analyses, the PNC assay was able to predict upcoming disease manifestations (within 2 months) in patients with SLE, including mucocutaneous features, alopecia, and glomerulonephritis. Altogether, the results of our investigation support the use of the PNC assay in clinical praxis to sharpen the diagnostics in undiagnosed patients and to improve the evaluation of prognosis in patients with SLE. Early identification of patients with negative prognostic factors, because of prediction of worsened outcome, should lead to earlier adjustment of their management and to overall decreased disease activity.

We started the investigation by assessing reference values of a PNC assay in apparently healthy individuals. The obtained reference values were evaluated in a Scandinavian group of patients with recent onset of any rheumatic symptoms. By comparing patients with SLE with patients affected by other disorders, we could demonstrate that SLE is by far the most common diagnosis related to a positive outcome of PNC assay, especially in anti-dsDNA antibody–positive patients (Table 1). One reason for a positive PNC assay result in patients with no SLE diagnosis and absence of detectable anti-dsDNA antibodies may be the presence in serum of autoantibodies against other parts of the NCM, including histones or nucleosomes.

In a realistic diagnostic setting, the physician is initially unaware of the correct diagnosis in patients with recent onset of rheumatic symptoms and the use of reliable laboratory tools plays an important diagnostic role. It is important to emphasize that the PPV for SLE after a cross-sectional analysis of the PNC assay is low. Other clinical diagnoses can be found in patients with a positive test, more frequently in anti-dsDNA–negative patients. We have previously reported that the PPV for SLE after a cross-sectional analysis of CLIFT alone in a similar population was 0.46 [32]. Nonetheless, the combined positivity of CLIFT and PNC assay in the present study resulted in a PPV of 0.67, suggesting an additive diagnostic role of the PNC assay in patients affected by SLE. Furthermore, although our study had a median follow-up of 4.7 years, it is possible that some of the individuals with positive PNC assay will still develop SLE in the future with or without any other overlapping rheumatic disease.

The second main aim of the present study was to investigate any potential predictive value of the PNC assay with regard to SLE-related clinical manifestations. In particular, we wanted to study whether the positive outcome of the test (alone or in combination with concomitant presence of anti-histone and/or anti-dsDNA antibodies and/or hypocomplementemia) coincides with or predicts the occurrence of any relevant clinical manifestations or changes in disease activity.

On one hand, the occurrence of glomerulonephritis was much more frequent in patients with SLE with persistent positive outcome of PNC assay, compared with those where the outcome was variable over time or always negative (Table 5). On the other hand, the occurrence of arthritis was much more frequent in patients with persistently negative PNC assay outcomes (Table 5). Although patients with PNC positivity had an increased SLEDAI-2K score, we could not show any significant association over time of the PNC assay with SLE disease activity. However, in combination with low complement levels, PNC positivity could predict upcoming flare, in particular active glomerulonephritis. The additive effect of low complement levels may be counterintuitive, considering the essential role of complements in the PNC assay [30]. However, low complement levels are often sufficient to activate the classical pathway of complements [33] and may also be a sign of ongoing immune complex–mediated disease, such as the ones detected in the PNC assay. Interestingly, positivity in the PNC assay also displayed an inverse association with development of arthritis, a clinical manifestation with much higher prevalence in patients with SLE with consistently negative PNC assay outcomes. It strongly suggests and supports the hypothesis that neutrophil abnormalities are related to a distinct lupus phenotype associated with severe manifestations, including hematological features and glomerulonephritis, similar to what has been described previously [30].

The PNC assay may help the physician to distinguish between different phenotypes in patients with SLE. The test displayed a highly significant correlation with concomitant hematological manifestations such as leukopenia and thrombocytopenia. It may suggest a potential role of the PNC assay as a marker for these relevant features. We do not have sufficient data at the moment to establish whether the hematological manifestations in our SLE cohort were related to active SLE or other causes, such as concomitant treatment with cytotoxic drugs, infections, or other. If our results are confirmed, the PNC assay could be a valid test that would provide the clinician with important information on whether the hematological manifestations are SLE-related. SLE-related hematological manifestations may require higher doses of immunosuppressive treatment, whereas a dose reduction or interruption of the treatment is often recommended whenever leukopenia and thrombocytopenia are supposed to be side effects of the treatment itself. To our knowledge, no other validated tests used in the management of patients with SLE are good enough to obtain the same information.

Limitations of the present investigation are mostly due to the cross-sectional analysis of the PNC assay in MRG patients and healthy individuals. Moreover, the laboratory analysis in MRG patients was limited to ANA and anti-dsDNA antibodies, besides the PNC assay. We have no data at the moment to discern anything about the variation over time of the outcome of the PNC test in healthy individuals or in patients without SLE with recent onset of rheumatic symptoms. This is an important aspect to analyze in future studies because the longitudinal analysis in the SLE cohort showed that the outcome of the PNC assay may influence the prevalence of crucial clinical features. Data that verify how anti-histone antibodies and the classical pathway of the complement system may influence the outcome of the PNC assay in these patients should also be included.

Recent discoveries, including the formation of neutrophil extracellular traps (NETs), a neutrophil cell death process in which several key autoantigens (including dsDNA and histones) are extruded from the neutrophil [34], as well as the identification of spontaneously NET-forming low-density granulocytes, an immature inflammatory neutrophil subset highly enriched in inflammatory conditions such as SLE [9, 10], have highlighted neutrophils as important immune cells in SLE pathogenesis [9, 11, 35]. Although NETs are cleared silently by macrophages in healthy individuals [36], patients with SLE have impaired clearance of NETs due to the presence of autoantibodies as well as decreased DNase I activity [6, 12, 37], enabling recognition of NETs by plasmacytoid dendritic cells and induction of type I interferons, key cytokines in SLE pathogenesis [38]. However, the clinical utility of assessing neutrophil function in the diagnosis and prognosis of patients with SLE has not been addressed. In the present study, we evaluated the diagnostic and prognostic value of the PNC assay, a functional test used to assess the ability of PMNs to engulf autoantibody- and complement-opsonized cell debris.

## Conclusions

Assessment of serum-mediated phagocytosis of NCM by PMNs as determined by the PNC assay may be considered a useful supplementary laboratory tool in the management of patients affected by SLE. Either alone or in combination with other laboratory tests, the PNC assay may provide the clinician with additional information in the diagnostic process and in assessing current and, more importantly, risk for future disease activity, as well as occurrence of clinical manifestations and phenotypes, such as glomerulonephritis, hematological, and mucocutaneous manifestations. Long-term longitudinal assessment of PNC assay in larger cohorts of patients with SLE is needed to validate the test as a relevant tool in the management of the disease.

## Abbreviations

ACR: American College of Rheumatology
ANA: anti-nuclear antibodies
CI: confidence interval
CLIFT: *Crithidia luciliae* immunofluorescence test
C1q: complement component C1q
C3: complement component C3
C4: complement component C4
dsDNA: double-stranded DNA
FcγRIIA: Fc γ-receptor IIA
FcγRIIIB: Fc γ-receptor IIIB
FSC-A: forward scatter analysis
LE: lupus erythematosus
LR: likelihood ratio
MRG: multicenter rheumatic group
NCM: necrotic cell material
NET: neutrophil extracellular trap
OR: odds ratio
PBMC: peripheral blood mononuclear cell
PI: propidium iodide
PMN: polymorphonuclear leukocyte
PNC: phagocytosis of necrotic cell material
PPV: positive predictive value
SD: standard deviation
Se: sensitivity
SLE: systemic lupus erythematosus
SLEDAI-2K: Systemic Lupus Erythematosus Disease Activity Index 2000
Sp: specificity
SSC-A: side scatter analysis
TLR: Toll-like receptor

## References

[1] Sanchez E, Rasmussen A, Riba L, Acevedo-Vasquez E, Kelly JA, Langefeld CD (2012). Impact of genetic ancestry and sociodemographic status on the clinical expression of systemic lupus erythematosus in American Indian-European populations. Arthritis Rheum.

[2] Crispin JC, Hedrich CM, Tsokos GC (2013). Gene-function studies in systemic lupus erythematosus. Nat Rev Rheumatol.

[3] Kanta H, Mohan C (2009). Three checkpoints in lupus development: central tolerance in adaptive immunity, peripheral amplification by innate immunity and end-organ inflammation. Genes Immun.

[4] Garcia-Romo GS, Caielli S, Vega B, Connolly J, Allantaz F, Xu Z (2011). Netting neutrophils are major inducers of type I IFN production in pediatric systemic lupus erythematosus. Sci Transl Med.

[5] Lande R, Ganguly D, Facchinetti V, Frasca L, Conrad C, Gregorio J (2011). Neutrophils activate plasmacytoid dendritic cells by releasing self-DNA-peptide complexes in systemic lupus erythematosus. Sci Transl Med.

[6] Leffler J, Gullstrand B, Jonsen A, Nilsson JA, Martin M, Blom AM (2013). Degradation of neutrophil extracellular traps co-varies with disease activity in patients with systemic lupus erythematosus. Arthritis Res Ther..

[7] Leffler J, Martin M, Gullstrand B, Tyden H, Lood C, Truedsson L (2012). Neutrophil extracellular traps that are not degraded in systemic lupus erythematosus activate complement exacerbating the disease. J Immunol.

[8] Smith CK, Kaplan MJ (2015). The role of neutrophils in the pathogenesis of systemic lupus erythematosus. Curr Opin Rheumatol.

[9] Denny MF, Yalavarthi S, Zhao W, Thacker SG, Anderson M, Sandy AR (2010). A distinct subset of proinflammatory neutrophils isolated from patients with systemic lupus erythematosus induces vascular damage and synthesizes type I IFNs. J Immunol.

[10] Hacbarth E, Kajdacsy-Balla A (1986). Low density neutrophils in patients with systemic lupus erythematosus, rheumatoid arthritis, and acute rheumatic fever. Arthritis Rheum.

[11] Villanueva E, Yalavarthi S, Berthier CC, Hodgin JB, Khandpur R, Lin AM (2011). Netting neutrophils induce endothelial damage, infiltrate tissues, and expose immunostimulatory molecules in systemic lupus erythematosus. J Immunol.

[12] Hakkim A, Furnrohr BG, Amann K, Laube B, Abed UA, Brinkmann V (2010). Impairment of neutrophil extracellular trap degradation is associated with lupus nephritis. Proc Natl Acad Sci U S A.

[13] Muñoz LE, Janko C, Schulze C, Schorn C, Sarter K, Schett G (2010). Autoimmunity and chronic inflammation—two clearance-related steps in the etiopathogenesis of SLE. Autoimmun Rev.

[14] Cohen AS, Reynolds WE, Franklin EC, Kulka JP, Ropes MW, Shulman LE (1971). Preliminary criteria for the classification of systemic lupus erythematosus. Bull Rheum Dis..

[15] Hochberg MC (1997). Updating the American College of Rheumatology revised criteria for the classification of systemic lupus erythematosus. Arthritis Rheum.

[16] Petri M, Orbai AM, Alarcon GS, Gordon C, Merrill JT, Fortin PR (2012). Derivation and validation of the Systemic Lupus International Collaborating Clinics classification criteria for systemic lupus erythematosus. Arthritis Rheum.

[17] Tan EM, Cohen AS, Fries JF, Masi AT, McShane DJ, Rothfield NF (1982). The 1982 revised criteria for the classification of systemic lupus erythematosus. Arthritis Rheum.

[18] Bertsias GK, Pamfil C, Fanouriakis A, Boumpas DT (2013). Diagnostic criteria for systemic lupus erythematosus: has the time come?. Nat Rev Rheumatol.

[19] Hargraves MM, Richmond H, Morton R (1948). Presentation of two bone marrow elements; the tart cell and the L.E. cell. Mayo Clin Proc.

[20] Feierl E, Smolen JS, Karonitsch T, Stummvoll GH, Ekhart H, Steiner CW (2007). Engulfed cell remnants, and not cells undergoing apoptosis, constitute the LE-cell phenomenon. Autoimmunity.

[21] Munoz LE, Janko C, Grossmayer GE, Frey B, Voll RE, Kern P (2009). Remnants of secondarily necrotic cells fuel inflammation in systemic lupus erythematosus. Arthritis Rheum.

[22] Holman S (1951). The lupus erythematosus cell inclusion phenomenon. J Clin Pathol.

[23] Rothfield NF, Pace N (1962). Relation of positive L.E.-cell preparations to activity of lupus erythematosus and corticosteroid therapy. N Engl J Med.

[24] Zimmermann-Górska I (1981). LE phenomenon in the synovial fluid in rheumatoid arthritis [in Polish]. Reumatologia.

[25] Schleissner LA, Sheehan WW, Orselli RC (1976). Lupus erythematosus in a patient with amyloidosis, adrenal insufficiency, and subsequent immunoblastic sarcoma: demonstration of the LE phenomenon in the lung. Arthritis Rheum.

[26] Lee SL, Michael SR, Vural IL (1951). The L.E. (lupus erythematosus) cell; clinical and chemical studies. Am J Med.

[27] Louis J, Limarzi LR (1958). The L. E. phenomenon and systemic lupus erythematosus. J Chronic Dis.

[28] Schett G, Steiner G, Smolen JS (1998). Nuclear antigen histone H1 is primarily involved in lupus erythematosus cell formation. Arthritis Rheum.

[29] Böhm I (2004). Flow cytometric analysis of the LE cell phenomenon. Autoimmunity.

[30] Gullstrand B, Lefort MH, Tyden H, Jonsen A, Lood C, Johansson A (2012). Combination of autoantibodies against different histone proteins influences complement-dependent phagocytosis of necrotic cell material by polymorphonuclear leukocytes in systemic lupus erythematosus. J Rheumatol.

[31] Gladman DD, Ibañez D, Urowitz MB (2002). Systemic Lupus Erythematosus Disease Activity Index 2000. J Rheumatol.

[32] Compagno M, Jacobsen S, Rekvig OP, Truedsson L, Heegaard NH, Nossent J (2013). Low diagnostic and predictive value of anti-dsDNA antibodies in unselected patients with recent onset of rheumatic symptoms: results from a long-term follow-up Scandinavian multicentre study. Scand J Rheumatol.

[33] Lood C, Gullstrand B, Truedsson L, Olin AI, Alm GV, Rönnblom L (2009). C1q inhibits immune complex–induced interferon-α production in plasmacytoid dendritic cells: a novel link between C1q deficiency and systemic lupus erythematosus pathogenesis. Arthritis Rheum.

[34] Brinkmann V, Reichard U, Goosmann C, Fauler B, Uhlemann Y, Weiss DS (2004). Neutrophil extracellular traps kill bacteria. Science.

[35] Knight JS, Zhao W, Luo W, Subramanian V, O’Dell AA, Yalavarthi S (2013). Peptidylarginine deiminase inhibition is immunomodulatory and vasculoprotective in murine lupus. J Clin Invest.

[36] Farrera C, Fadeel B (2013). Macrophage clearance of neutrophil extracellular traps is a silent process. J Immunol.

[37] Leffler J, Ciacma K, Gullstrand B, Bengtsson AA, Martin M, Blom AM (2015). A subset of patients with systemic lupus erythematosus fails to degrade DNA from multiple clinically relevant sources. Arthritis Res Ther..

[38] Rönnblom L (2011). The type I interferon system in the etiopathogenesis of autoimmune diseases. Ups J Med Sci.

